# COVID-19 Progression: A County-Level Analysis of Vaccination and Case Fatality in Mississippi, USA

**DOI:** 10.3390/ijerph192416552

**Published:** 2022-12-09

**Authors:** Shinjita Ghosh, Hafiz A. Ahmad, Luma Akil, Paul B. Tchounwou

**Affiliations:** 1 Department of Biology, NIH-RCMI Center for Health Disparities Research, Jackson State University, Jackson, MS 39217, USA; 2 Department of Behavioral and Environmental Health, School of Public Health, Jackson State University, Jackson, MS 39217, USA

**Keywords:** COVID-19, socioeconomic-determinants of health, health disparities, Mississippi

## Abstract

The COVID-19 pandemic has created a severe upheaval in the U.S., with a particular burden on the state of Mississippi, which already has an exhausted healthcare burden. The main objectives of this study are: (1) to analyze the county-level COVID-19 cases, deaths, and vaccine distribution and (2) to determine the correlation between various social determinants of health (SDOH) and COVID-19 vaccination coverage. We analyzed COVID-19-associated data and county-level SDOH factors in 82 counties of Mississippi. The cumulative COVID-19 and socio-demographic data variables were grouped into feature and target variables. The statistical and exploratory data analysis (EDA) was conducted using Python 3.8.5. The correlation between the target and feature variables was performed by Pearson Correlation analysis. The heat Map Correlation Matrix was visually presented to illustrate the correlation between each pair of features and each target variable. Results indicated that people of Asian descent had the highest vaccination coverage of 77% fully vaccinated compared to 52%, 46%, 42% and 25% for African Americans, Whites, Hispanics, and American Indians/Alaska Natives, respectively. The county-level vaccination rate was significantly higher among the minority populations than the White population. It was observed that COVID-19 cases and deaths were positively correlated with per capita income and negatively correlated with the percentage of persons without a high school diploma (age 25+). This study strongly demonstrates that different SDOH factors influence the outcome of the COVID-19 vaccination rate, which also affects the total number of COVID-19 cases and deaths. Vaccine promotion should be given to all populations regardless of race and ethnicity to achieve uniform acceptance. Therefore, statewide policy recommendations focusing on specific community needs should help achieve health equity in COVID-19 vaccination management.

## 1. Introduction

COVID-19 is a global health catastrophe that has changed the entire health dynamics of human society. It has eventually led to a drastic shift in the political, economic, and social responses worldwide [1]. COVID-19 is a form of pulmonary disease first reported in Wuhan (Hubei Province, China) on 31 December 2019, and is caused by a novel coronavirus (2019-nCoV), labeled as SARS-CoV-2 [2].

SARS-CoV-2 is highly contagious and easily transmissible in proximity from one person to another. As of 20 April 2022, the total number of COVID-19 cases in the United States stands at 80,526,422, and the total number of COVID-19 deaths is 986,545 [3]. COVID-19 incidence rate in the US is 24,322 per 100,000 population. The COVID-19 outbreak highlights the vulnerability to novel infections, and vaccination remains one of the most reliable methods to return to normal life [4]. CDC recommends different preventive measures to prevent the spread of the COVID-19 pandemic, such as wearing masks or respirators, increasing space and distance between two people, mostly in indoor settings, improving indoor air ventilation, increasing outdoor activities, getting tested for COVID-19 when suspected of probable infection, following health regulatory authorities recommendations in case of probable suspecting of COVID-19 infection, staying home when you have confirmed or suspected COVID-19 infections and seeking appropriate treatment for COVID-19, etc. [5]. Additionally, proper selection of surface disinfectants, adequate hand sanitization, and increased usage of PPE are potential interventions to fight against COVID-19. They are proven beneficial in stopping the spreading of the disease [6]. These recommended measures are hugely impactful in preventing the overall spread of COVID-19 across society. Alongside these recommendations, scientists, pharmaceutical and biotech industries, and government authorities focus on developing and distributing vaccines against COVID-19 as the only measure to protect the entire population from this deadly pandemic [7]. COVID-19 vaccines are beneficial and effective in preventing severe disease, hospitalization, and death from COVID-19. In the US, everyone aged five and older are eligible to be vaccinated [8]. Three COVID-19 vaccines manufactured by Pfizer-BioNTech, Moderna and Johnson & Johnson are available in the US. Pfizer-BioNTech and Moderna are messenger RNA (mRNA) vaccines that use comparatively new technology. Johnson & Johnson is a carrier, or virus vector, a vaccine that has been used before for the flu [9].

In the US, 66% population is now fully vaccinated, whereas, in Mississippi, only 52% are fully vaccinated. United States has one of the fastest COVID-19 vaccine rollouts globally; however, disparities are also prominent in vaccination coverage amongst the entire population [3,10,11]. Different social determinants of health (SDOH) factors influence certain groups of people differently, resulting in health disparities in vaccine acceptance and availability.

Human health is impacted by diverse factors, which are categorized as follows: genetic, behavioral, environmental, physical and medical care, and social factors. The fifth category (social factors) is also referred to as SDOH; it comprises economic and social circumstances that influence health [12]. As per Healthy People 2030, SDOHs are grouped into five different domains that, include: financial stability, education access, and quality, health care access and quality, neighborhood and build environment, and the social and community context that affect a wide range of health, functioning, and quality-of-life outcomes and risks [13].

A recent study using over 3000 U.S. counties with various demographic, environmental, and socioeconomic factors found that socioeconomic factors strongly correlate with COVID-19 vaccination rates and incidence rates, underscoring the need to improve COVID-19 vaccination campaigns in marginalized communities [14]. In another study by [7], US counties with low insurance coverage are associated with higher COVID-19 incidence rates and slower vaccine rollout. It is a well-known factor that vaccine hesitancy is among the primary issues in the vaccination campaign [8]. The Centers for Diseases Control (CDC) has conducted a study on Patterns in COVID-19 Vaccination Coverage by Social Vulnerability and Urbanicity in the United States [15]. It showed that COVID-19 vaccination coverage disparities by Social Vulnerability Index (SVI) have continued and amplified over time, even as vaccination eligibility and access have expanded. A review of different journal articles, newspaper editorials, and blogs reveals that a robust vaccination-associated health disparity is prominent in the US [16]. However, none of the studies has solely focused on COVID-19 cases, deaths, and vaccination rates analysis in Mississippi, a state with a large minority population and a high poverty level.

Mississippi has the worst economic hardship index of all 50 states, which compares state economic conditions based on crowded housing, dependency, education, income, poverty, and unemployment [17]. The general population tends to congregate in counties with higher economic opportunities, thus concentrating on people with a high per capita income and education. Given that COVID-19 is highly contagious, it is likely to affect more in those counties where people are clustered for economic and employment reasons [18].

The vaccination trend is influenced by various socioeconomic factors that may or may not be the same for total COVID-19 cases and deaths in Mississippi. The present study aims to analyze the county-level COVID-19 cases, deaths, and vaccine distribution and determine the correlation between various social determinants of health (SDOH) and COVID-19 vaccination coverage. No ecological studies have been published yet focusing on COVID-19 cases, deaths, and vaccination distributions, solely focusing on the state of Mississippi. Therefore, such a country-level analysis of total COVID-19 cases, deaths, and vaccination distribution in Mississippi to understand the SDOH correlation will enhance our understanding of this deadly pandemic in the least prosperous state of the US.

## 2. Materials and Methods

### 2.1. Data Collection

The present study analyzed COVID-19-associated data and county-level SDOH factors in 82 counties of Mississippi that have cumulative COVID-19 data available through 3 March 2022. COVID-19 total cases, deaths, and Mississippi vaccination data were collected from the CDC and the Mississippi State Department of Health [2,3,19]. The County level SDOH factors or the county-level social and environmental variables for the state of Mississippi were obtained from the CDC, Mississippi State Department of Health, Agency for Toxic Substances and Disease Registry, National Center for Health Statistics, and United States Census Bureau [2,3,4,10,11,20].

### 2.2. Data Term and Variable Description

Total COVID-19 cases are defined as the total number of COVID-19 cases since the beginning of the pandemic in the entire state of Mississippi. Total COVID-19 deaths are defined as the total number of COVID-19 deaths since the inception of the pandemic in Mississippi. The vaccination rate in Mississippi is expressed as the percentage of individuals in the state fully vaccinated with COVID-19 vaccine doses (two doses of Pfizer/Moderna or one dose of the Johnson and Johnson vaccine).

Various SDOH factors contribute directly or indirectly to the health disparities in the US. The CDC/ATSDR SVI uses US Census data to determine the social vulnerability of every census tract. The CDC/ATSDR SVI ranks each tract on 15 social factors, including poverty, lack of vehicle access, and crowded housing, and groups them into four related themes. Each track receives a separate ranking for the four themes and an overall order [21]. Amongst them, the county-level SDOH factors or county-level social and environmental variables used in this study are the percentage of persons below the poverty estimate, unemployment rate estimate, per capita income estimate, 2014–2018 ACS, percentage of persons with no high school diploma (age 25+) estimate, percentage of persons aged 65 and older estimate, 2014–2018 ACS and percentage minority (all persons except white, non-Hispanic) estimate, 2014–2018 ACS, urban-rural classification scheme for counties, social vulnerability index (SVI) scores and COVID-19 community vulnerability index (CCVI) scores. Both SVI scores and CCVI scores vary from 0 to 1; lower scores represent lower vulnerability, and higher scores represent higher vulnerability.

The COVID-19 CCVI is computed using 40 measures within seven themes: socioeconomic status (SES); minority status and language; housing type, transportation, household composition, and disability (“housing type/composition” hereafter); epidemiological factors; healthcare system factors; high-risk environments; and population density [8].

### 2.3. Statistical Analysis

For the correlation analysis, the study variables were divided into the following two categories:

**Features Variables**

1. **EP_POV:** Percentage of persons below the poverty estimate.
2. **EP_UNEMP:** Unemployment Rate estimate.
3. **EP_PCI:** Per capita income estimate, 2014–2018 ACS.
4. **EP_NOHSDP:** Percentage of persons with no high school diploma (age 25+) estimate.
5. **EP_AGE65**: Percentage of persons aged 65 and older estimate, 2014-2018 ACS.
6. **EP_MINRTY:** Percentage minority (all persons except white, non-Hispanic) estimate, 2014–2018 ACS.
7. **EP_UNINSUR:** Percentage of uninsured in the total civilian noninstitutionalized population estimate, 2014–2018 ACS.
8. **Urban-Rural Classification**: 2013 NCHS urban-rural classification scheme for counties.
9. **CDC SVI:** Social vulnerability index (SVI) scores, which range from 0 to 1.
10. **CCVI:** COVID-19 community vulnerability index (CCVI) scores, which also vary from 0 to 1, are from Surgo Ventures.

**Target Variables**

1. Total COVID-19 cases in Mississippi (as of 3 March 2022).
2. Total COVID-19 deaths in Mississippi (as of 3 March 2022).
3. Full Vaccinated Population in Mississippi (as of 3 March 2022).

The statistical analysis and exploratory data analysis (EDA) for this study were conducted using Python 3.8.5 Version in the jupyter notebook under the Google Colaboratory cloud computing system. Python libraries like Pandas, Numpy, Seaborn, and Matplotlib were used for the entire analysis.

A Pearson correlation analysis was conducted to determine the correlation between the target and feature variables. The null hypothesis is as follows:

1. No correlation between target and feature variables is established (independence).
2. The target and feature variables are statistically correlated (dependence).

The significance level was set at 0.05; any variables with a *p*-value < 0.05 reject the null hypothesis and accept a strong to a medium correlation between the said two variables.

The Pearson correlation method identifies the association between feature variables and target variables. Pearson correlations were computed for each county-level feature as follows:

1. The feature of interest and county-level vaccination rate.
2. The feature of interest and county-level total cases.
3. The feature of interest and county-level death rate.

### 2.4. Data Visualization

The data were visually represented using the Heat Map Correlation Matrix (HMCM). HMCM represents the Pearson correlation plot of county-level features variables compared with target variables. Each square shows the correlation between the variables on each axis. Correlation ranges from −1 to +1. The diagonals are all 1/white because those squares correlate each variable to itself (so it’s a perfect correlation). For the rest, the larger the number and darker the color, the higher the correlation between the two variables. The plot is also symmetrical about the diagonal since the same two variables are paired together in those squares.

## 3. Results

**Figure 1:**
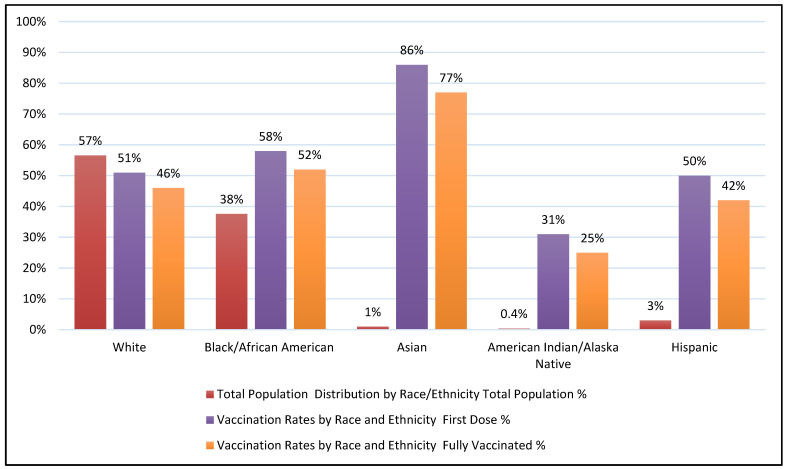
Vaccination administration amongst the Mississippi population based on race and ethnicity. Notes: Population and demographic data are based on the Census Bureau’s American Community Survey (ACS) analysis and may differ from other population estimates published yearly by the Census Bureau. US Mississippi state vaccination data were obtained from the Mississippi State Department of Health website. The vaccination data included one dose and a fully vaccinated percentage. Native Hawaiian or Other Pacific Islander, two or more races, unknown races, and Unknown ethnicity peoples are excluded from the chart.

In Mississippi, 1,537,240 people (52%) were fully vaccinated; 81% population of ≥65 years of age and 52% population of ≥12 years of age were vaccinated entirely. Figure 1 presents an essential representation of the vaccination administration scenario in the Mississippi population based on their race and ethnicity. The figure shows that the Mississippi population comprises 57% Whites, 38% African Americans, 3% Hispanics, 1% Asians, and 0.4% American Indians and Alaska Natives. The data also indicate that while people of Asian descent represent only 1% of the Mississippi population, they have the highest vaccination coverage, with 77% of them fully vaccinated compared to 52%, 46%, 42%, and 25% for African Americans, Whites, Hispanics, and American Indians/Alaska Natives, respectively.

**Figure 2:**
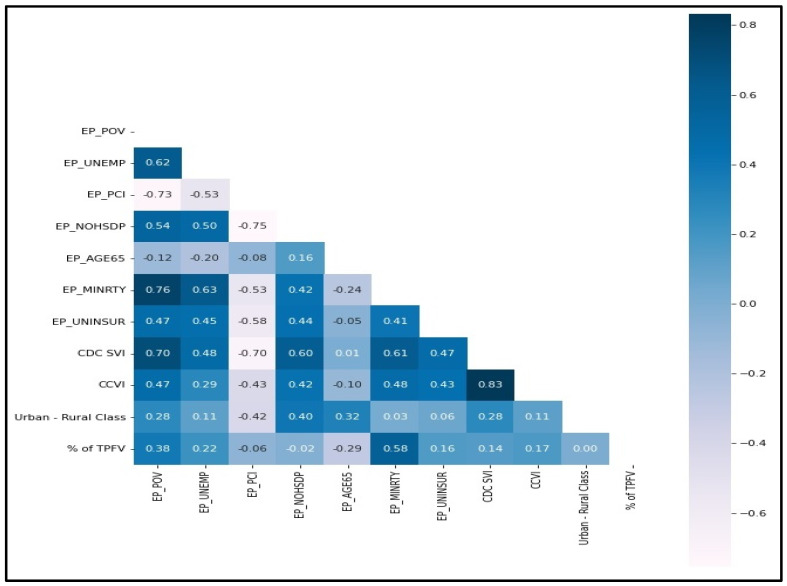
Heat Map Correlation Matrix of County-Level Features for County-Level Vaccination Rate.

A correlation between county-level features and county-level vaccination rate was represented in Figure 2 as Heat Map Correlation Matrix. The Pearson correlation value for the feature vs. vaccination rate is shown for each county-level feature. Variable correlation values > 0.5 represent a strong to a medium correlation between two variables. It is observed that EP_MINRTY, i.e., percentage of minorities (all persons except white, non-Hispanic) variable, is strongly and positively correlated with vaccination rate. There is a medium positive correlation between the percentage of persons below the poverty rate and the unemployment rate with county-level vaccination rate. So, these two factors did not make a big difference in total vaccine distribution in Mississippi.

**Figure 3:**
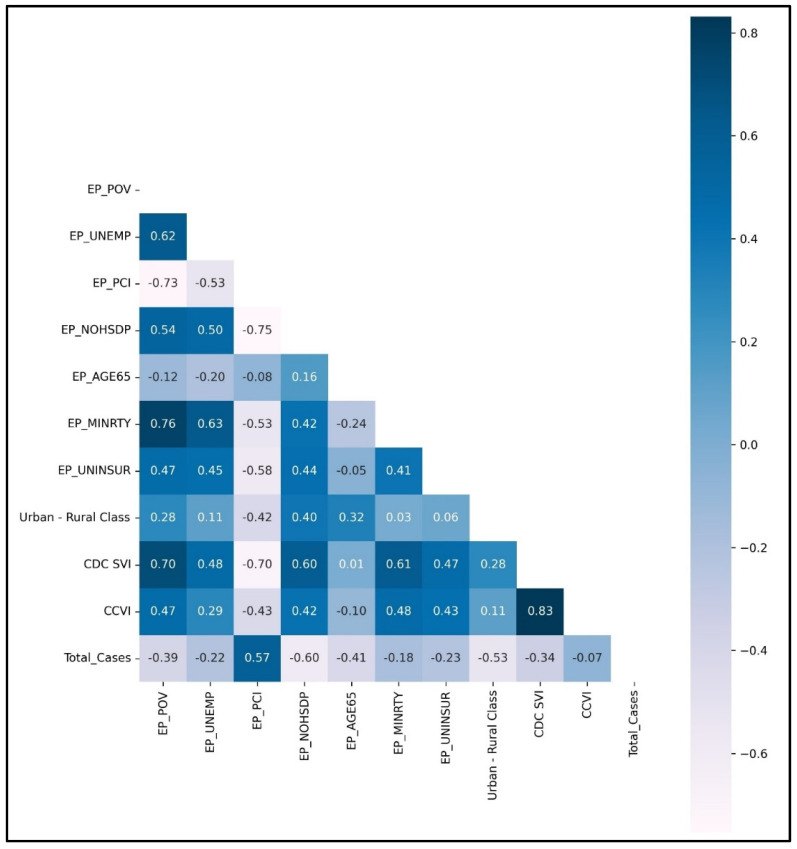
Heat Map Correlation Matrix of County-Level Features for County-Level Total Cases (Cumulative COVID-19 cases).

The correlation between county-level features and county-level total cases (COVID-19 total cases) was represented in Figure 3 as Heat Map Correlation Matrix. The EP_PCI, i.e., per capita income estimate variable, is positively related to COVID-19 total cases. It implies that Mississippi counties with a high per capita income have more cumulative COVID-19 total cases. It is also negatively correlated with EP_NOHSDP, i.e., the percentage of persons with no high school diploma (age 25+) estimate. It can be inferred that those counties where most people have a primary education have higher cumulative COVID-19 total cases. EP_PCI and EP_NOHSDP are inversely correlated because counties with a high per capita income have more populations with primary education and employment opportunities, indicating a higher population density than other counties. It is comprehensible that a highly contagious and infectious disease like COVID-19 is clustering in places with more people nearby.

**Figure 4:**
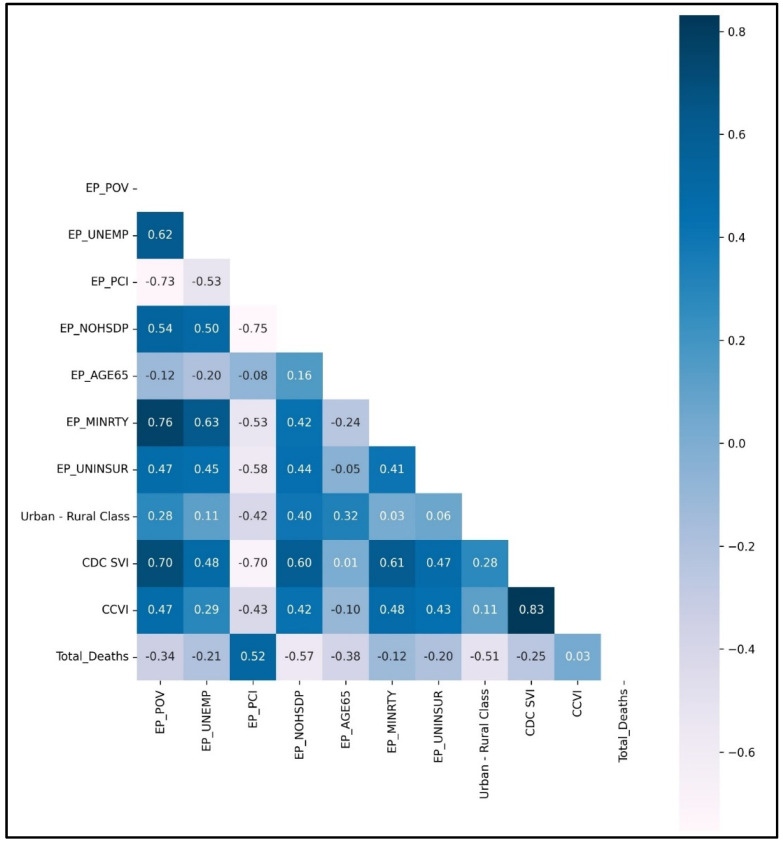
Heat Map Correlation Matrix of County-Level Features for County-Level Total Deaths (Cumulative COVID-19 Deaths).

A similar trend is also observed with cumulative COVID-19 total deaths, as represented in Figure 4 Heat Map Correlation Matrix. EP_PCI and EP_NOHSDP variables correlate with total COVID-19 deaths, similar to COVID-19 cases. COVID-19 and COVID-19 reflect a similar trend among the social variables influencing the outcome.

The urban-rural classification variable strongly correlates with COVID-19 total cases and total deaths. Cumulative COVID-19 cases and deaths are higher in urban counties due to the population with higher per capita income and a percentage of the population with higher education.

## 4. Discussion

The correlation values, *p*-values, and 95% CLvalues for all three target variables compared to the features variables are given in Table 1 to determine if similar SDOH variables had an impact. The results indicate that a variable such as EP_MINRTY, i.e., percentage of the minority population, is the main influencing factor in the vaccination rate in different counties of Mississippi. However, EP_MINRTY does not impact cumulative COVID-19 cases and deaths. Similarly, EP_PCI, EP_NOHSDP, and urban-rural classification are significant variables that affect total COVID-19 cases and deaths, and these three variables do not correlate with the total COVID-19 vaccination rate. The results of this study strongly reflect that influencing SDOH factors on the outcome of the COVID-19 vaccination rate is entirely different from those affecting total COVID-19 cases and deaths.

Different SDOH factors significantly affect the COVID-19 cases, deaths, and vaccination patterns in various counties of Mississippi. Total COVID-19 cases and deaths impact counties, where people are clustered for economic opportunities. Mississippi is a rural state, and agriculture is its primary industry; therefore, few counties where people are assembled for different occupational resources observe a faction of those populations with high education and high income compared to other counties. Such congregations trigger more COVID-19 cases and deaths in those counties than in others. The major SDOH factor affecting the Mississippi vaccination rate is EP_MINRTY, i.e., the percentage of minorities (all persons except white, non-Hispanic). Additionally, from Figure 1, it is visible that 86% of Asians are vaccinated. So, it is visible the percentage of full vaccination coverage is higher amongst Asian Americans than among other racial and ethnic groups, which is also a national trend [22]. However, Asian Americans share just 1% of the total population distribution. So, their higher vaccination coverage has minimal effect on Mississippi’s total population vaccination rate.

Studies have reported that economically and socially disadvantaged populations have been disproportionately affected by COVID-19–related morbidity and mortality [23]. Vaccination coverage was shown to be consistently lower in high-vulnerability counties than in low-vulnerability counties with low socioeconomic status, such as high poverty, unemployment, low income, and no high school diploma [24]. However, some states achieved higher COVID-19 vaccination coverage in high-vulnerability counties. Some of the practices that allowed to reach the high coverage included “(1) prioritizing persons in racial/ethnic minority groups during the early stages of the vaccine program implementation, (2) actively monitoring and addressing barriers to vaccination in vulnerable communities, (3) directing vaccines to vulnerable communities, (4) offering free transportation to vaccination sites, and (5) collaborating with community partners, tribal health organizations, and the Indian Health Service” [24,25]. Some of these practices may help disadvantaged states such as MS achieve higher vaccination rates among the minority population.

Hence, the above discussion gives us a clear statistic of COVID-19 cases, deaths, and vaccine distribution status in Mississippi. The major SDOH factors that affect the COVID-19 vaccination coverage in MS are also identified and discussed. The study is entirely grounded on retrospective data analysis of collected data which always differ from the ground-level present scenario and different microstructural influential factors which are not covered in the captured data. There is also a gap in data as it is impossible to collect all other branches of SDOH factors affecting different parameters of COVID-19 diseases without proper public health program planning; these are some of the study’s significant limitations.

## 5. Conclusions

COVID-19-related safety measures should be increased in counties with a high population density. As the disease is highly infectious amongst people in close proximity, additional safety measures are required in places where people are clustered for occupational reasons, which may not be otherwise avoidable by simple safety guidelines. Different population or zip code analyses of these counties show a high correlation with COVID-19 cases and deaths. These analyses could be beneficial in understanding their population distribution and various social vulnerability factors that may impact the COVID-19 outbreak. The present study indicates that several social vulnerability factors also influence the full vaccination coverage in Mississippi. An emphasis on different public health program planning requirements is also visible, which will provide ground for more in-depth data collection and research work. Public health programs will also be beneficial for improving COVID-19-associated social backlogs and necessities. Emphasis should be given to vaccine promotion amongst all populations to achieve a coherent vaccine acceptance result regardless of race and ethnicity.

Another major factor for vaccine promotion is social media. In the current era, social media dramatically impacts disease trends and preventive measures acceptancy. A study result suggests that using social media to devour news content can increase vaccine hesitancy by cumulating citizens’ skepticism regarding the efficacy of vaccines. However, these effects depend upon users’ news literacy, as the impact on vaccine hesitancy is more substantial among those with lower news literacy. That research study article recommends to public policymakers and vaccine communication strategists that any attempt to reduce vaccine hesitancy in society should factor in the adverse effects of social media news use that can increase vaccine safety concerns [26].

Hence, the findings from this study could provide a helpful course of action for policymakers to develop specific programs for improving the vaccination rate throughout the state of Mississippi. Additionally, statewide policy recommendations focusing on specific community-based needs will help achieve health equity in COVID-19 vaccination management. Additionally, statewide policy recommendations focusing on specific community-based needs will help achieve health equity in COVID-19 vaccination management.

## Figures and Tables

**Table 1:** Pearson Correlation Value of COVID-19 Target Variables compared to SDOH Feature Variables.

County-Level Feature	Vaccination Rate Correlation Value	Vaccination Rate 95% Cl	Vaccination Rate *p*-Value	Total Cases Correlation Value	Total Cases 95% Cl	Total Cases *p*-Value	Total Deaths Correlation Value	Total Deaths 95% Cl	Total Deaths *p*-Value
EP_POV	0.3797	[0.18, 0.55]	<0.05	−0.394	[−0.56, −0.19]	<0.05	−0.3413	[−0.52, −0.13]	<0.05
EP_UNEMP	0.223	[0.01, 0.42]	<0.05	−0.2177	[−0.42, −0.0]	<0.05	−0.2083	[−0.41, 0.01]	>0.05
EP_PCI	−0.0637	[−0.28, 0.16]	>0.05	0.5728	[0.41, 0.7]	<0.05	0.5216	[0.34, 0.66]	<0.05
EP_NOHSDP	−0.0181	[−0.23, 0.2]	>0.05	−0.5978	[−0.72, −0.44]	<0.05	−0.5674	[−0.7, −0.4]	<0.05
EP_AGE65	−0.2859	[−0.47, −0.07]	<0.05	−0.413	[−0.58, −0.22]	<0.05	−0.3825	[−0.55, −0.18]	<0.05
EP_MINRTY	0.5784	[0.41, 0.71]	<0.05	−0.1767	[−0.38, 0.04]	>0.05	−0.1224	[−0.33, 0.1]	>0.05
EP_UNINSUR	0.1567	[−0.06, 0.36]	>0.05	−0.2267	[−0.42, −0.01]	<0.05	−0.1984	[−0.4, 0.02]	>0.05
CDC SVI	0.1412	[−0.08, 0.35]	>0.05	−0.3399	[−0.52, −0.13]	<0.05	−0.2535	[−0.45, −0.04]	>0.05
CCVI	0.1652	[−0.05, 0.37]	>0.05	−0.0721	[−0.28, 0.15]	>0.05	0.0311	[−0.19, 0.25]	>0.05
Urban-Rural Class	0.0045	[−0.21, 0.22]	>0.05	−0.5282	[−0.67, −0.35]	<0.05	−0.5068	[−0.65, −0.33]	<0.05

## Data Availability

All data generated from this research are presented in this manuscript.
